# “The Stress of the Situation”: How Do Compounding Experiences of Oppression Impact Emotional Distress Among a Diverse Sample of Internally Displaced People in Colombia?

**DOI:** 10.1192/bjo.2022.204

**Published:** 2022-06-20

**Authors:** Rachel Kerrigan, Rochelle Burgess, Laura Fonseca

**Affiliations:** 1 Institute of Global Health, University College London, London, United Kingdom; 2 Universidad de La Sabana, Chia, Colombia

## Abstract

**Aims:**

Addressing the mental health needs of Internally Displaced People (IDPs) in Colombia has been identified as a public health priority. Women and disabled IDPs are recognised as under-researched populations, with differences in vulnerabilities to displacement and resettlement prospects. This thematic network analysis employs an intersectional approach to consider *how* compounding experiences of oppression impact emotional distress among IDPs to enable informed and appropriate service provision.

**Methods:**

This is a qualitative analysis of a subset of data collected by the second author and her research team in 2017–18, as part of a larger action research project. Participants were randomly selected from the Victim's register in an industrialised municipality of Colombia. A subsample (n = 20) were invited to participate in life and family histories. Units of analysis were individual (n = 11) and family interviews (n = 9), with a mixture of self-identifying disabled and non-disabled men and women. River and tree of life tools were used to elicit culturally sensitive discussion of significant life events and ongoing distress. NVivo software and hand coding techniques were used to operationalise thematic webs. The analysis employed a grounded approach to thematic network analysis.

**Results:**

Three global themes, each underpinned by several organisational themes, were developed. The first, *Environments and contexts of displacement,* considers the loss of land and community alongside the myriad of social institutions, legal entitlements, family circumstances, cultural expectations and stigma influencing participants' access to resources. The second, *Making sense of it all*, represents the emotional and cognitive responses to perceived injustices and eroded trust. The third, *Mechanisms for managing distress*, represents strategies employed by IDPs at individual and family levels. Relationships between employment status and gendered divisions of labour were noted, suggesting that non-disabled women were able to meet increased domestic and paid work demands following displacement, though this was a considerable source of stress. Concepts around racial, indigenous and class identities were alluded to by several participants but could not be fully developed due to relative scarcity of accounts within the dataset.

**Conclusion:**

The thematic networks presented illustrate several compounding and interrelated oppressions faced by IDPs, offering explanation as to how this produces and sustains emotional distress. Participants’ well-founded worries about economic security and childcare alongside concerns for safety and acceptance in host communities require co-ordinated, locally informed responses. Prevention and recovery programmes should consider interventions at a family level, whilst strengthening participants’ self-developed strategies for managing distress.

